# Supplementary figures and images for: Transmission Dynamics of Zika Virus in Island Populations: A Modelling Analysis of the 2013–14 French Polynesia Outbreak
Source: PLoS Negl Trop Dis. 2016 May 17;10(5):e0004726. doi: 10.1371/journal.pntd.0004726 (PMC4871342; doi:10.1371/journal.pntd.0004726)

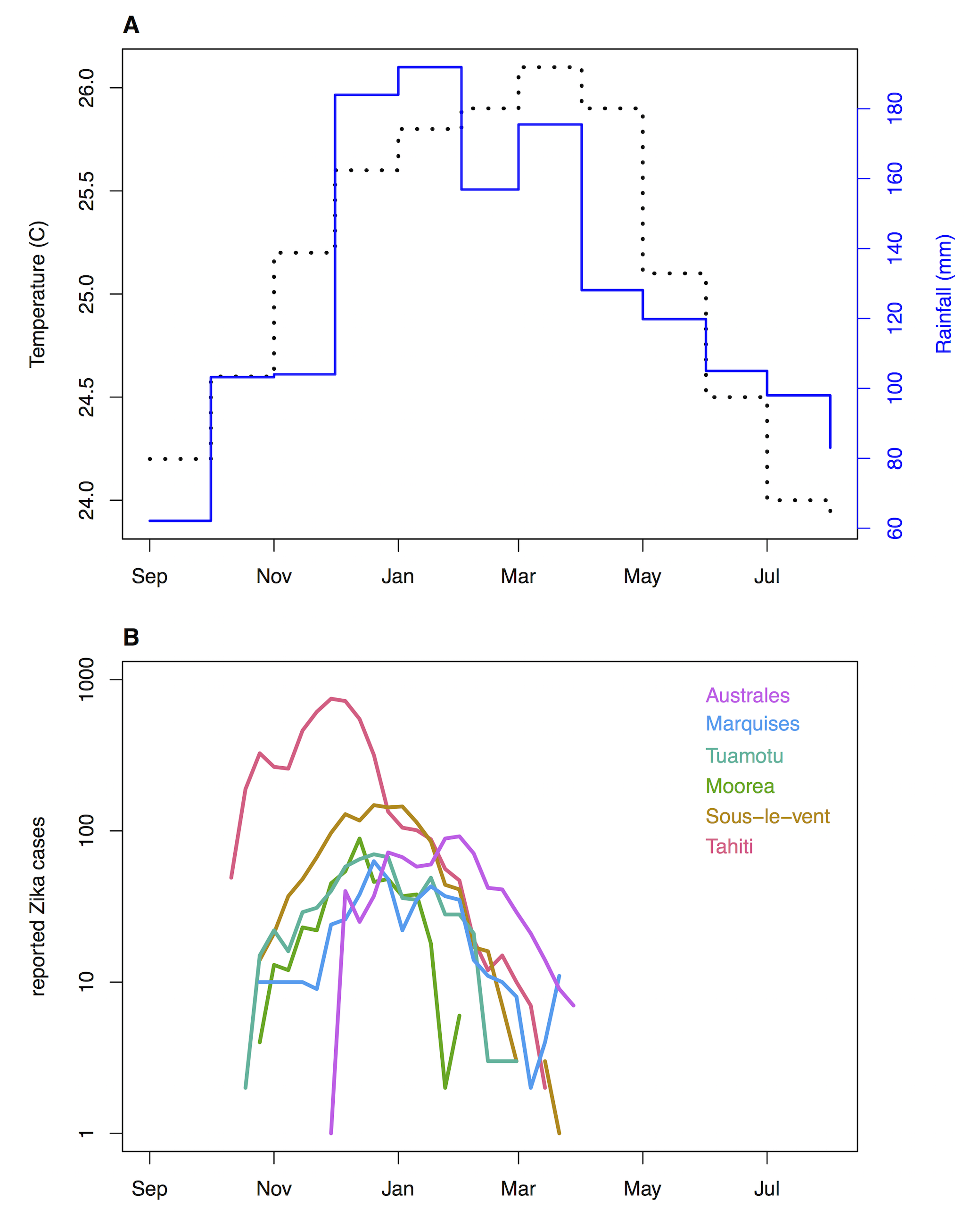

Supplement: S1 Fig — (A) Mean monthly temperature and rainfall in French Polynesia from 1990–2012. (B) Suspected ZIKV cases in 2013–14. (TIFF) [file pntd.0004726.s001.tiff]

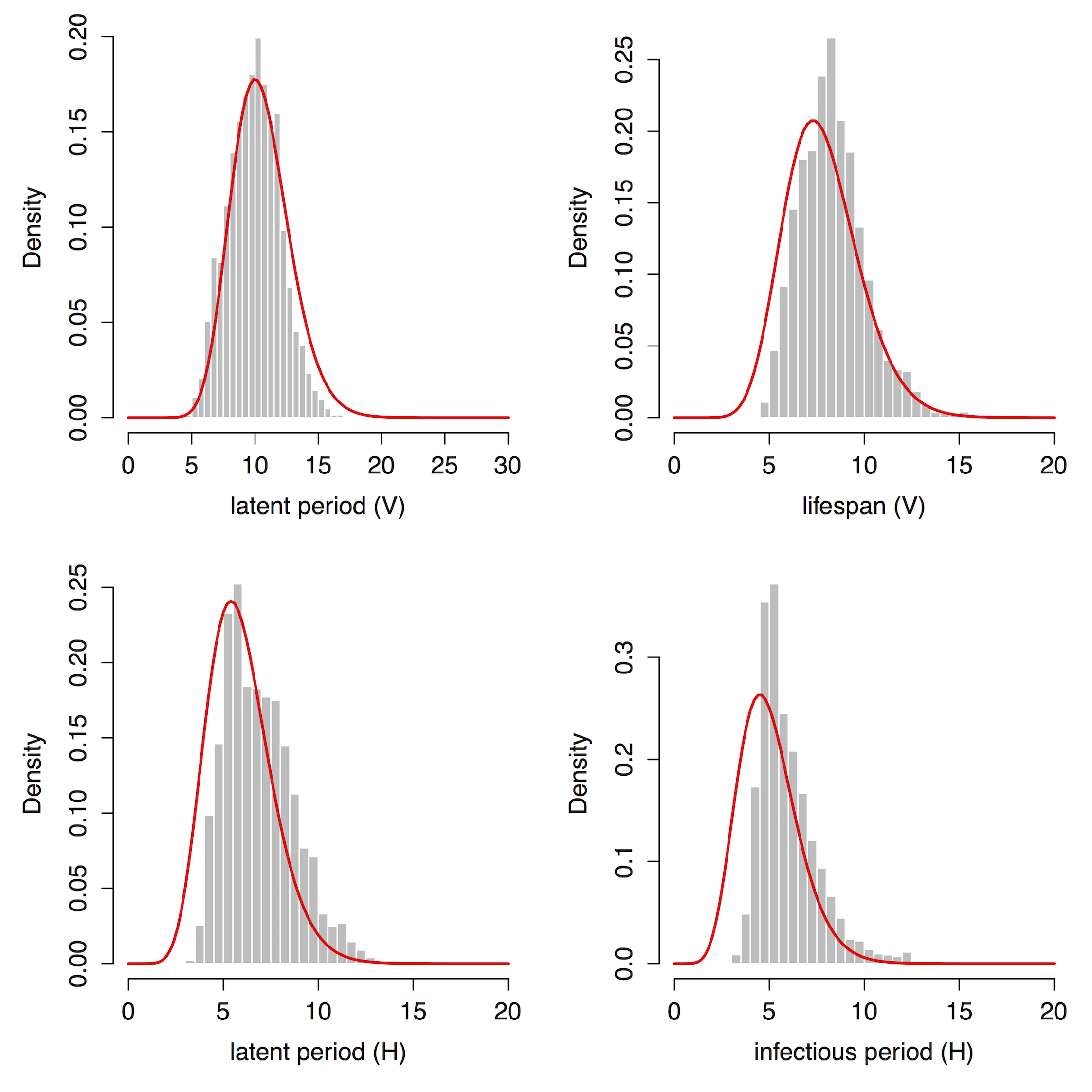

Supplement: S2 Fig — The parameters were jointly fitted across all six regions. (TIFF) [file pntd.0004726.s002.tiff]

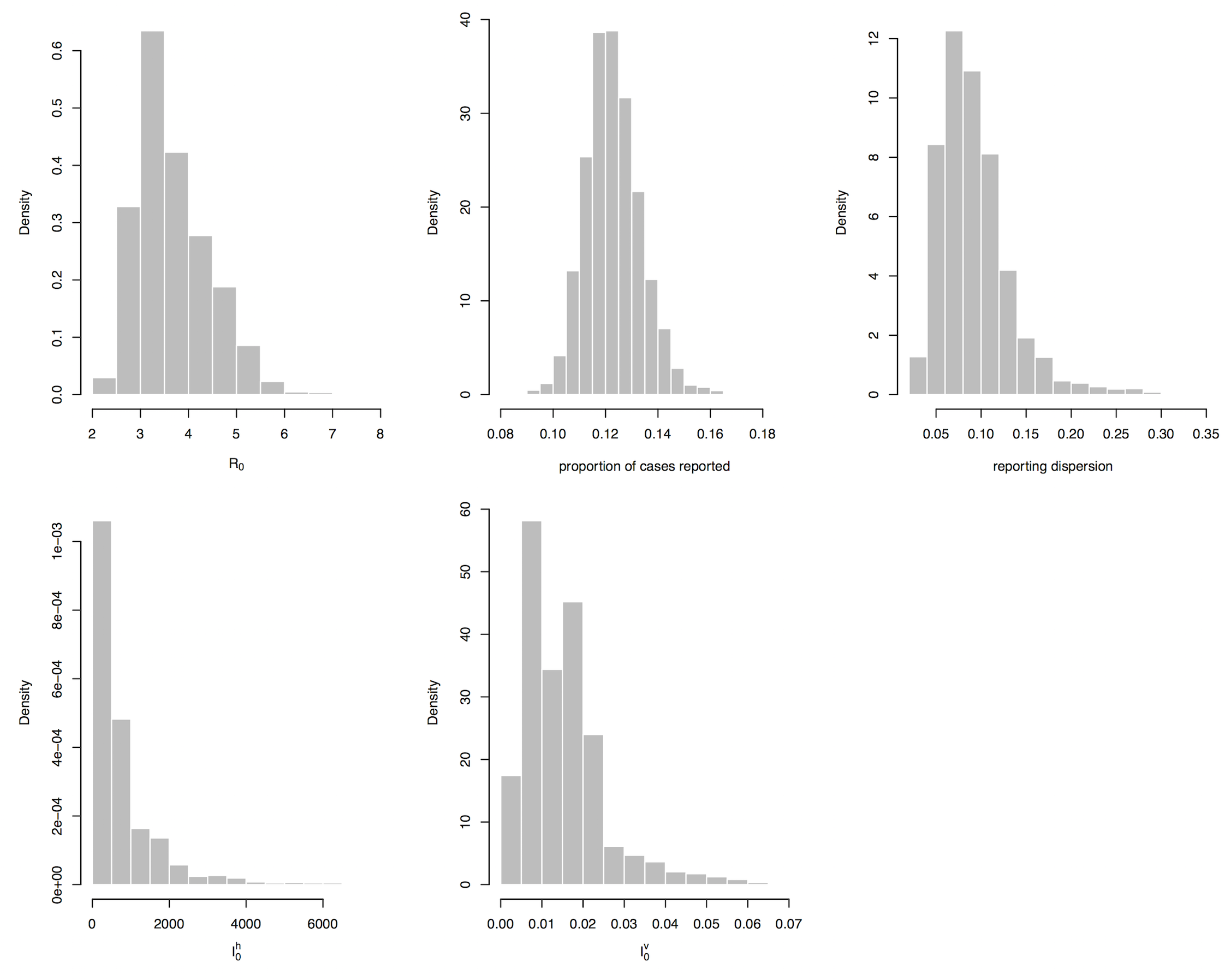

Supplement: S3 Fig — Plot shows marginal posterior estimates for: the basic reproduction number, R0; the proportion of cases reported, r; the dispersion parameter for the reporting distribution, ϕ; the number of initially infectious humans, I0H and the proportion of the mosquito population initially infectious, I0V. (TIFF) [file pntd.0004726.s003.tiff]

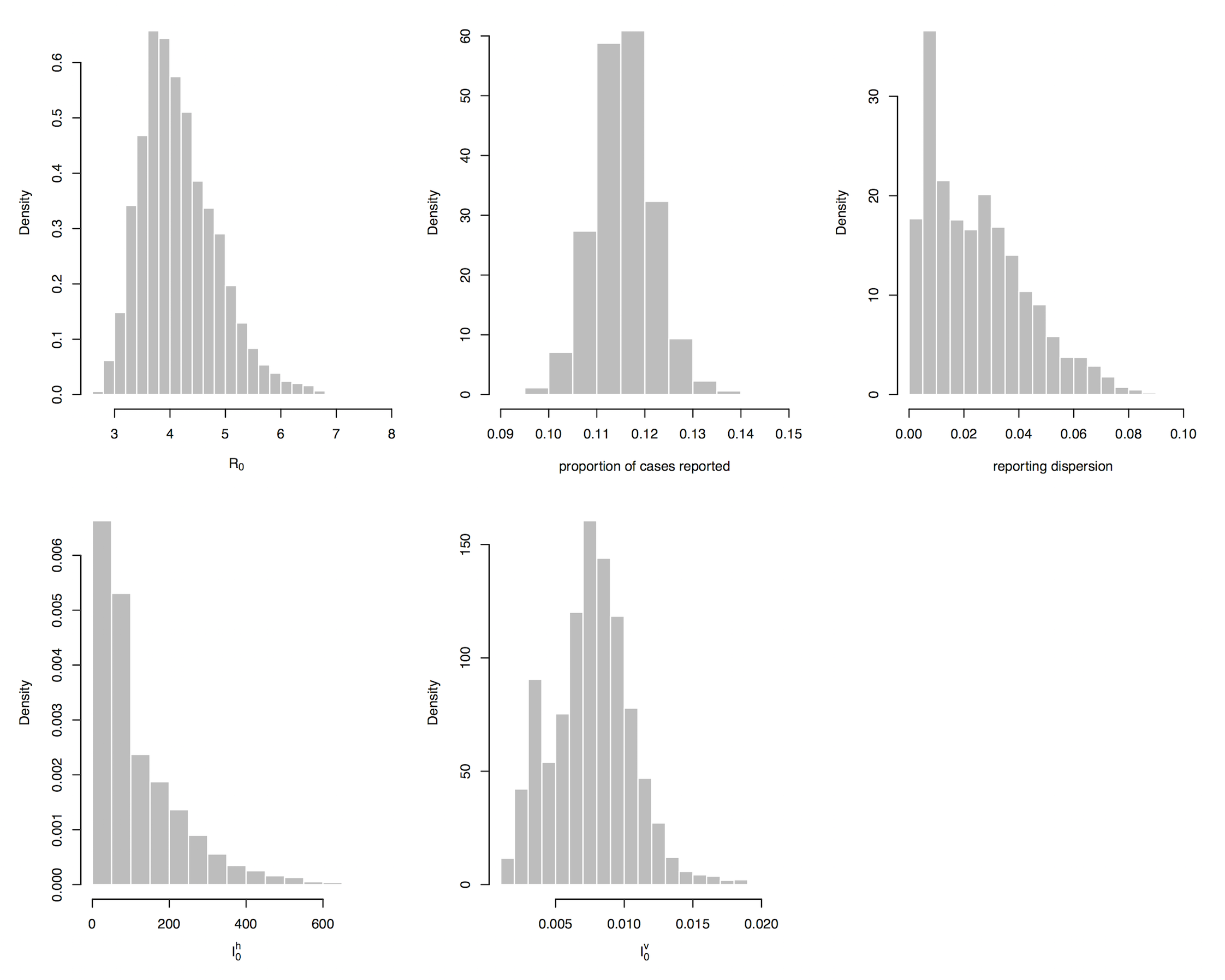

Supplement: S4 Fig — Plot shows marginal posterior estimates for: the basic reproduction number, R0; the proportion of cases reported, r; the dispersion parameter for the reporting distribution, ϕ; the number of initially infectious humans, I0H and the proportion of the mosquito population initially infectious, I0V. (TIFF) [file pntd.0004726.s004.tiff]

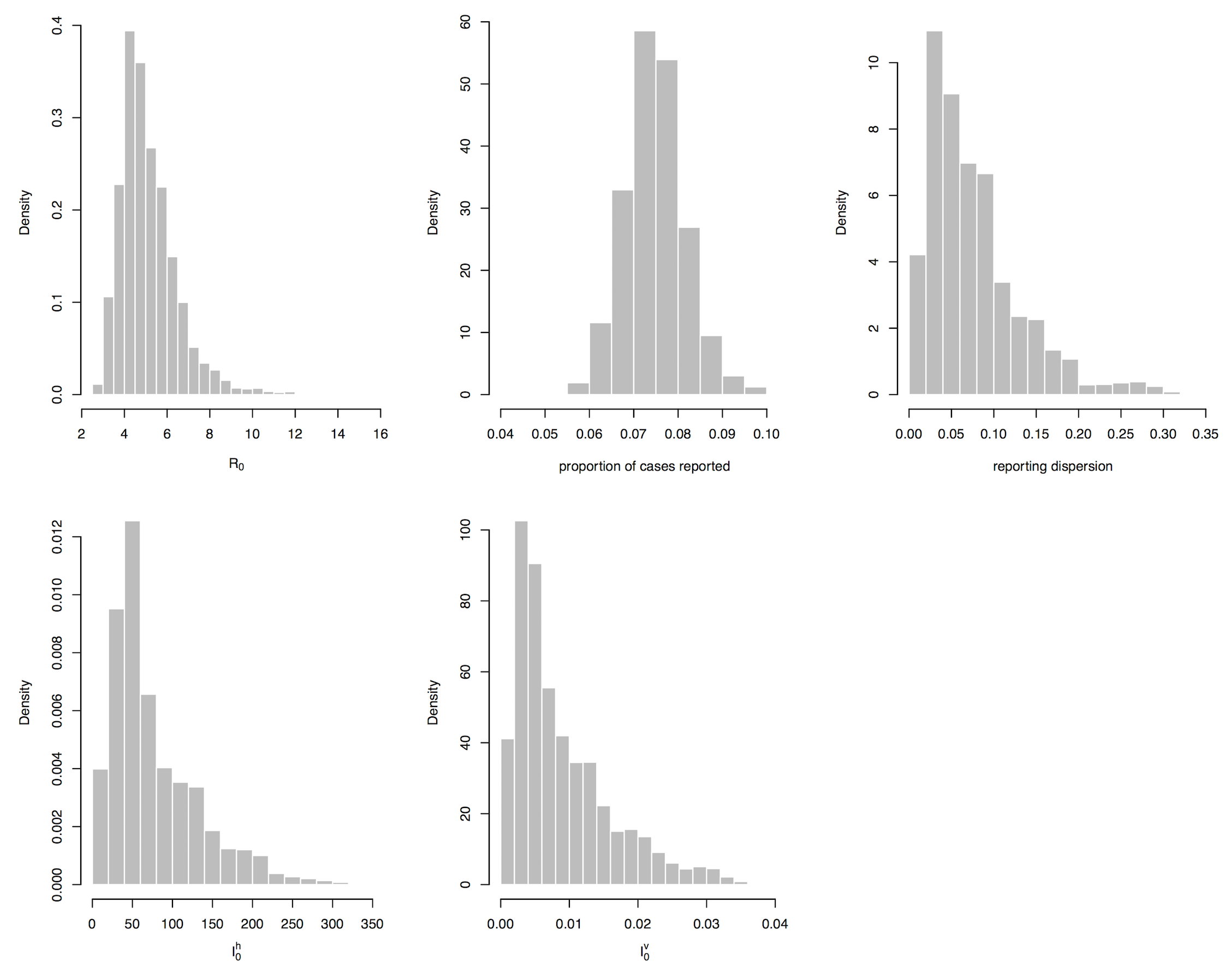

Supplement: S5 Fig — Plot shows marginal posterior estimates for: the basic reproduction number, R0; the proportion of cases reported, r; the dispersion parameter for the reporting distribution, ϕ; the number of initially infectious humans, I0H and the proportion of the mosquito population initially infectious, I0V. (TIFF) [file pntd.0004726.s005.tiff]

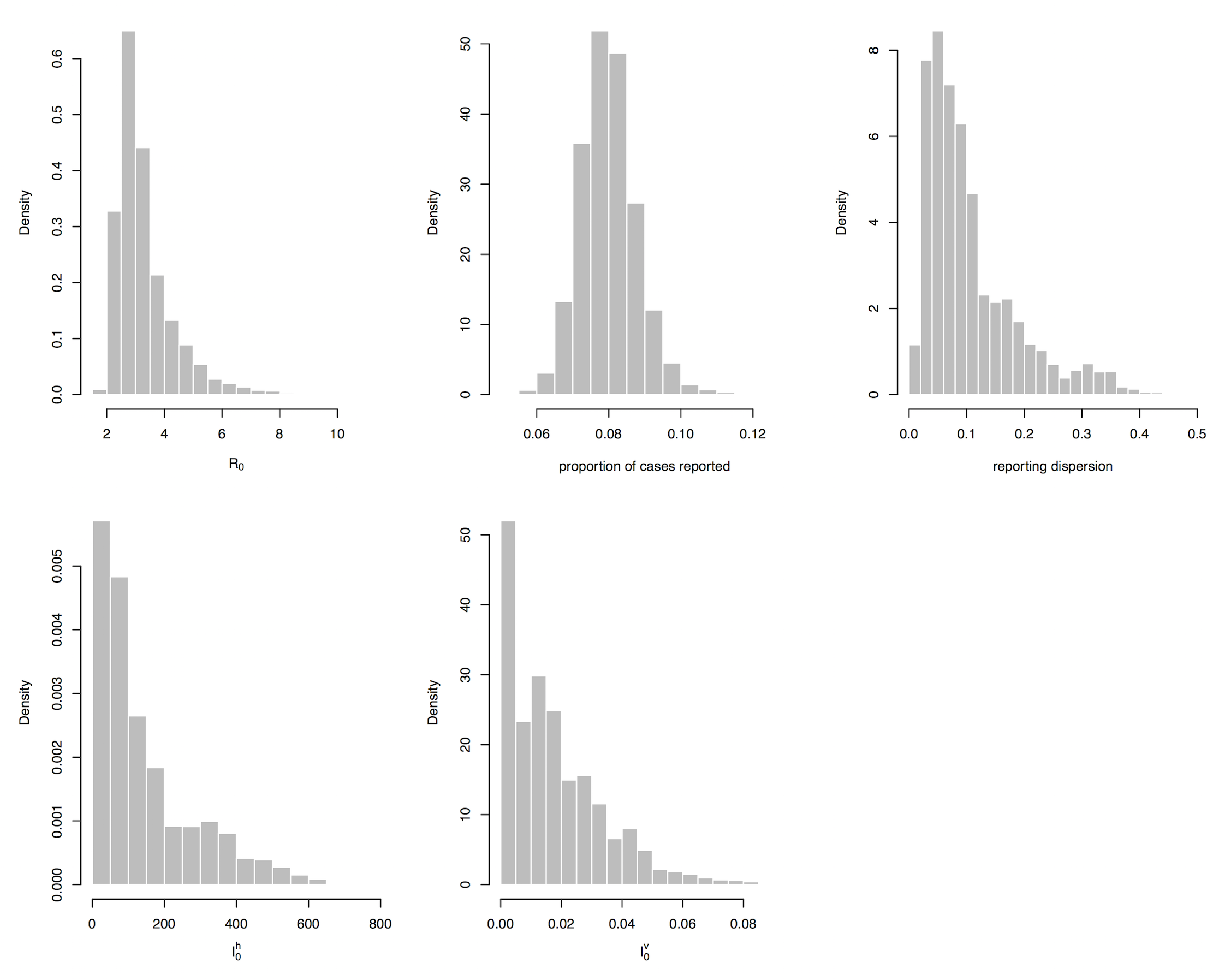

Supplement: S6 Fig — Plot shows marginal posterior estimates for: the basic reproduction number, R0; the proportion of cases reported, r; the dispersion parameter for the reporting distribution, ϕ; the number of initially infectious humans, I0H and the proportion of the mosquito population initially infectious, I0V. (TIFF) [file pntd.0004726.s006.tiff]

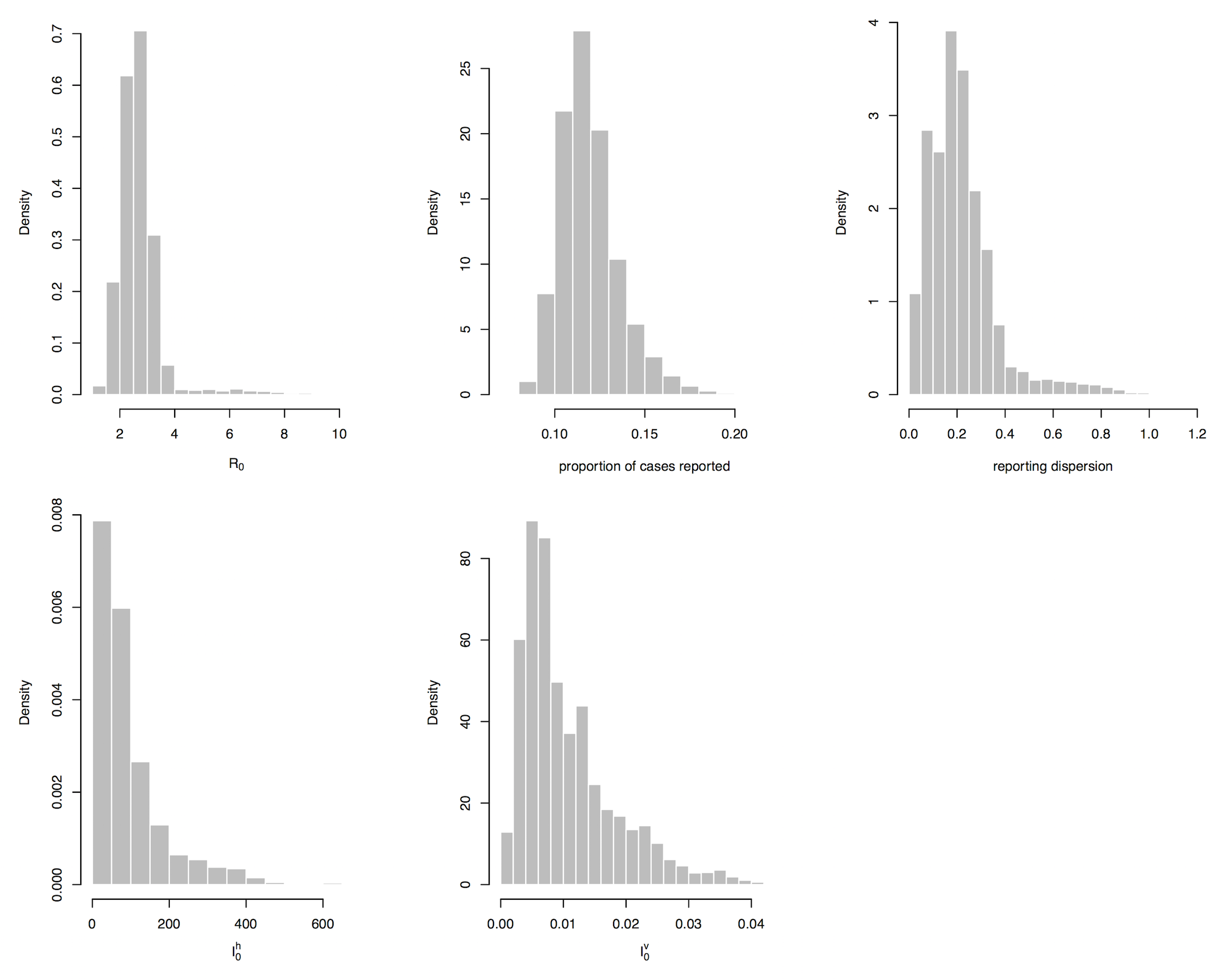

Supplement: S7 Fig — Plot shows marginal posterior estimates for: the basic reproduction number, R0; the proportion of cases reported, r; the dispersion parameter for the reporting distribution, ϕ; the number of initially infectious humans, I0H and the proportion of the mosquito population initially infectious, I0V. (TIFF) [file pntd.0004726.s007.tiff]

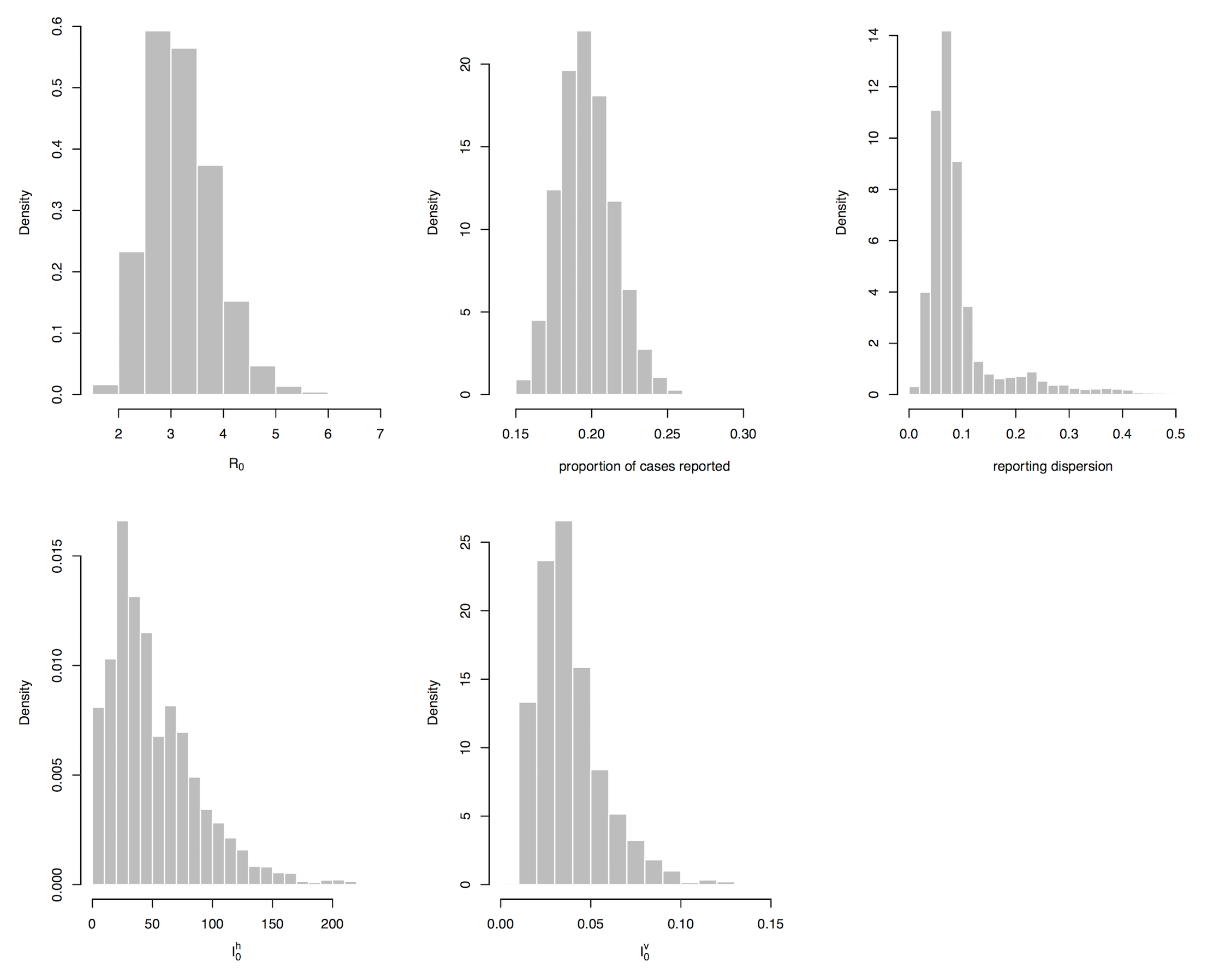

Supplement: S8 Fig — Plot shows marginal posterior estimates for: the basic reproduction number, R0; the proportion of cases reported, r; the dispersion parameter for the reporting distribution, ϕ; the number of initially infectious humans, I0H and the proportion of the mosquito population initially infectious, I0V. (TIFF) [file pntd.0004726.s008.tiff]

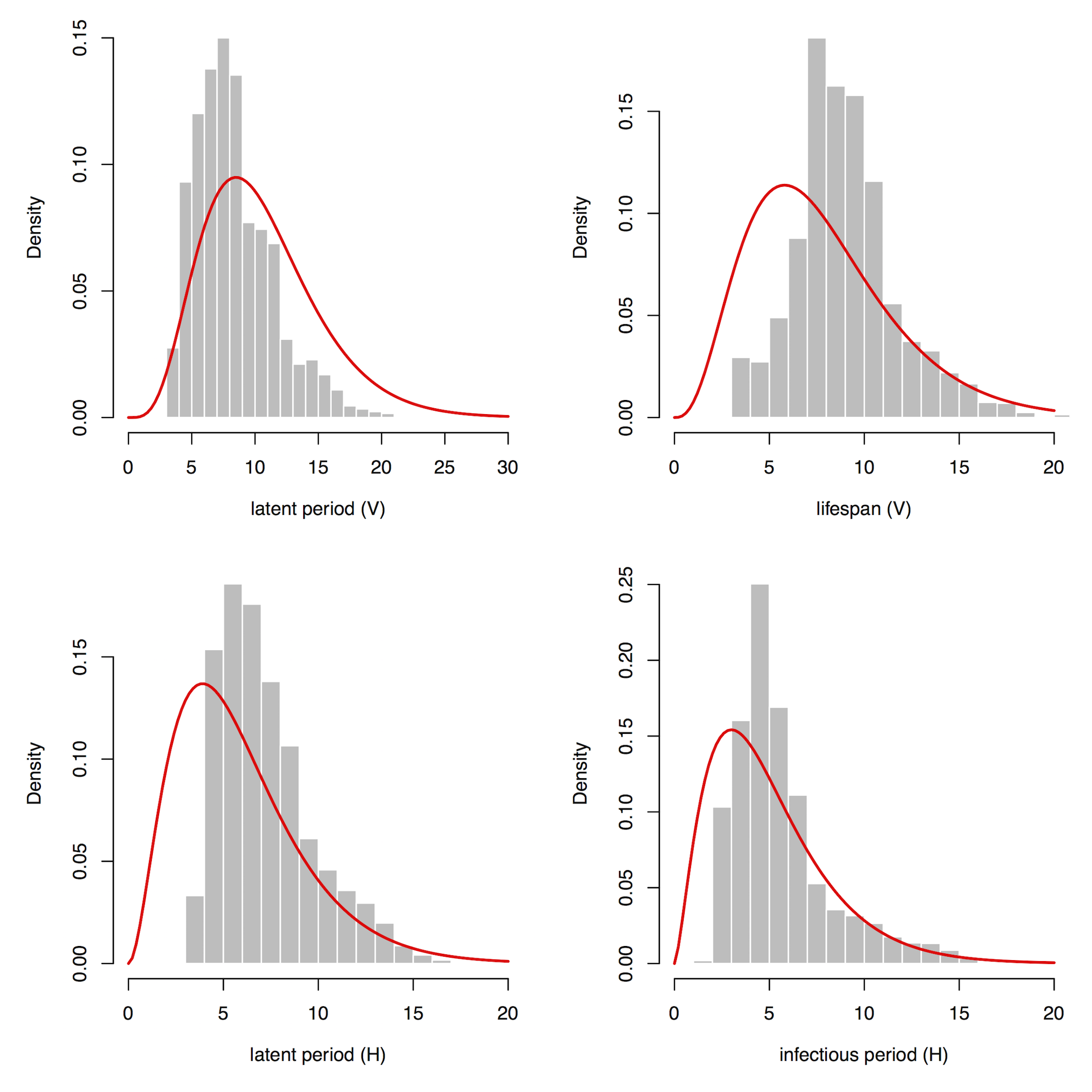

Supplement: S9 Fig — (TIFF) [file pntd.0004726.s009.tiff]

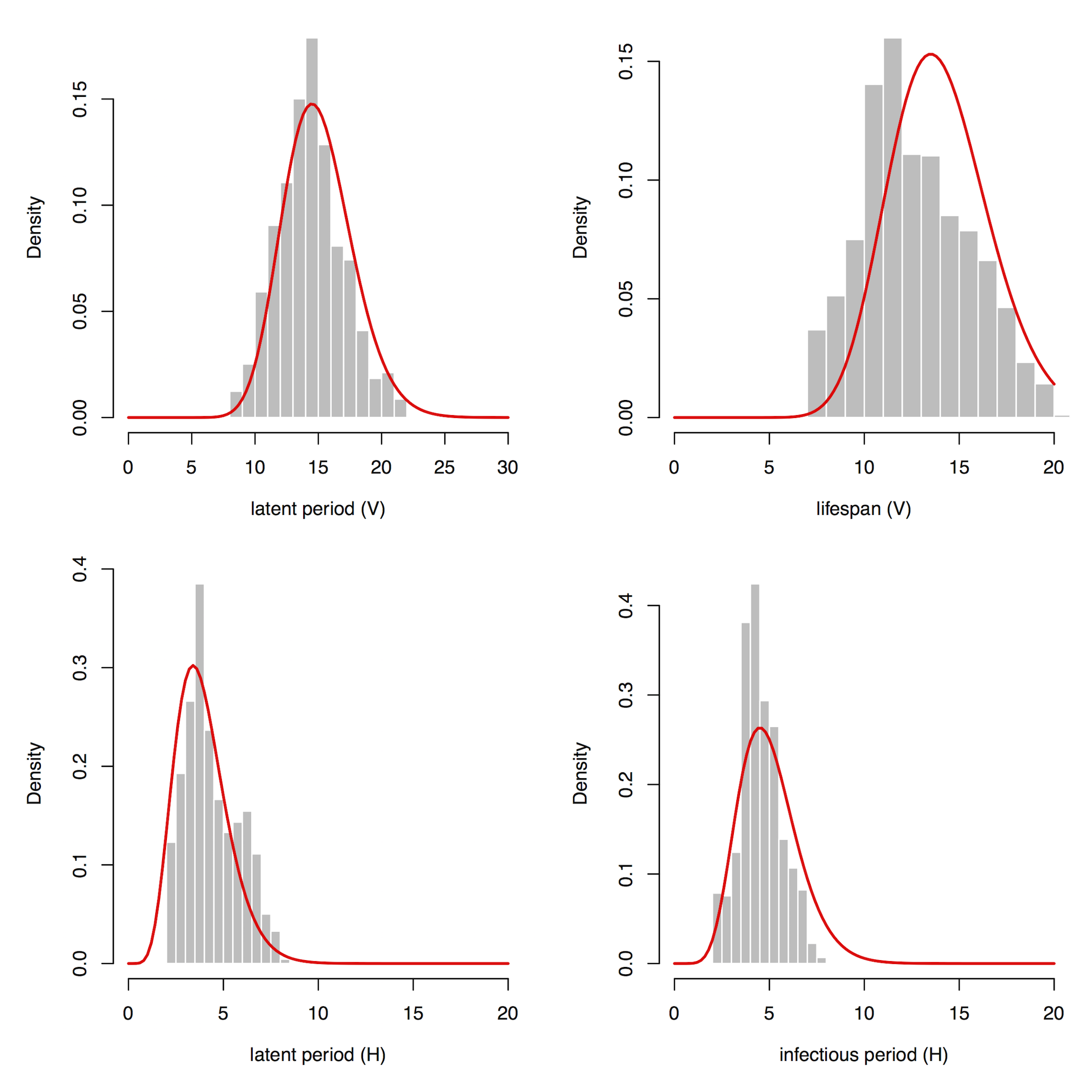

Supplement: S10 Fig — (TIFF) [file pntd.0004726.s010.tiff]

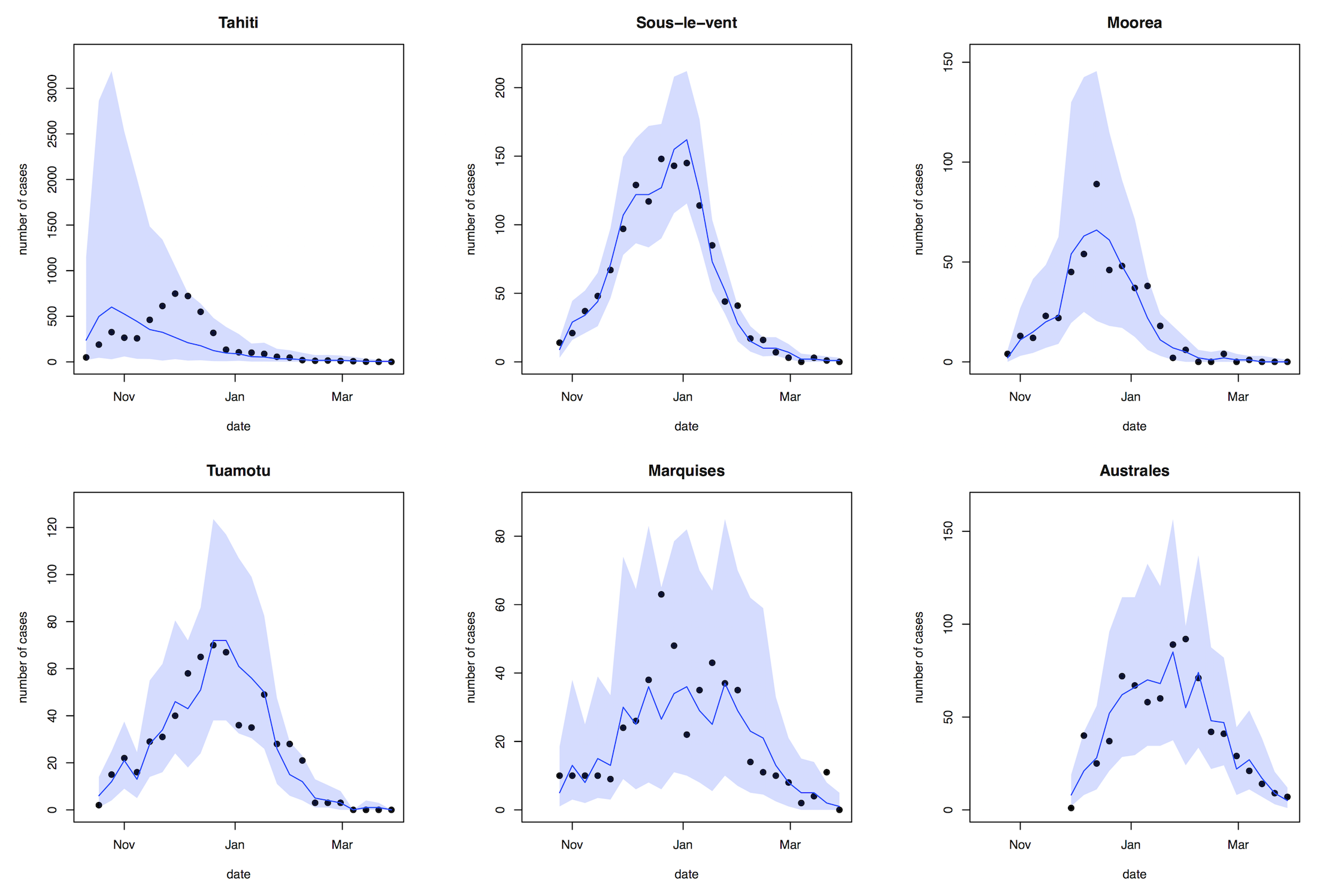

Supplement: S11 Fig — (TIFF) [file pntd.0004726.s011.tiff]
