# Supplementary material for: Transmission Dynamics of Zika Virus in Island Populations: A Modelling Analysis of the 2013–14 French Polynesia Outbreak
Source: PLoS Negl Trop Dis. 2016 May 17;10(5):e0004726. doi: 10.1371/journal.pntd.0004726 (PMC4871342; doi:10.1371/journal.pntd.0004726)
Supplement: S1 Table — Parameters are as described in Table 2. Median estimates are given, with 95% credible intervals in parentheses. Full posterior distributions are shown in S2–S8 Figs. (PDF) [file pntd.0004726.s012.pdf]

Table S1: **Estimated parameters for ZIKV infection.** Parameters are as described in Table 2. Median estimates are given, with 95% credible intervals in parentheses. Full posterior distributions are shown in Figures S2–S8.

Island	R_0	β_h	β_v	α_h	γ	α_v	δ	r	ϕ	$I_H(0)$	$I_V(0)$
Tahiti	3.5 (2.6-5.3)	0.57 (0.17-1.9)	0.27 (0.07-1.1)	6.6 (4.2-11)	5.6 (4-10)	10 (6.3-14)	8.2 (5.5-12)	0.12 (0.1-0.15)	0.085 (0.04-0.2)	450 (71-3500)	0.014 (0.0038-0.043)
Sous-le-vent	4.1 (3.1-5.7)	0.45 (0.24-1.1)	0.43 (0.16-1.1)	6.6 (4.2-11)	5.6 (4-10)	10 (6.3-14)	8.2 (5.5-12)	0.12 (0.1-0.13)	0.022 (0.0034-0.068)	82 (3-430)	0.0077 (0.0024-0.013)
Moorea	4.8 (3.2-8.4)	0.35 (0.16-1.2)	0.64 (0.15-1.6)	6.6 (4.2-11)	5.6 (4-10)	10 (6.3-14)	8.2 (5.5-12)	0.075 (0.062-0.089)	0.062 (0.0084-0.23)	58 (11-220)	0.0066 (0.0012-0.028)
Tuamotu-Gambier	3 (2.2-6.1)	0.34 (0.064-2)	0.42 (0.11-4.4)	6.6 (4.2-11)	5.6 (4-10)	10 (6.3-14)	8.2 (5.5-12)	0.08 (0.066-0.097)	0.082 (0.021-0.33)	92 (12-510)	0.014 (0.0012-0.058)
Marquises	2.6 (1.7-5.3)	0.52 (0.073-4.1)	0.22 (0.024-3.9)	6.6 (4.2-11)	5.6 (4-10)	10 (6.3-14)	8.2 (5.5-12)	0.12 (0.094-0.16)	0.19 (0.04-0.67)	64 (13-370)	0.0081 (0.002-0.03)
Australes	3.1 (2.2-4.6)	0.27 (0.11-0.66)	0.53 (0.2-2)	6.6 (4.2-11)	5.6 (4-10)	10 (6.3-14)	8.2 (5.5-12)	0.2 (0.16-0.24)	0.074 (0.029-0.33)	41 (5.2-140)	0.034 (0.014-0.086)
