# Supplementary material for: Transmission Dynamics of Zika Virus in Island Populations: A Modelling Analysis of the 2013–14 French Polynesia Outbreak
Source: PLoS Negl Trop Dis. 2016 May 17;10(5):e0004726. doi: 10.1371/journal.pntd.0004726 (PMC4871342; doi:10.1371/journal.pntd.0004726)
Supplement: S2 Table — Estimates for the basic reproduction number, R0; the proportion of infected individuals that were reported as suspected cases at sentinel sites; and the total proportion of the population infected (including both symptomatic and asymptomatic cases, with reports following a negative binomial distribution with reporting proportion r and dispersion parameter ϕ). Median estimates are given, with 95% credible intervals in parentheses. (PDF) [file pntd.0004726.s013.pdf]

Table S2: **Estimated parameters for ZIKV infection when prior distributions with $\sigma = 2$ are used.** Estimates for the basic reproduction number, R_0; the proportion of infected individuals that were reported as suspected cases at sentinel sites; and the total proportion of the population infected (including both symptomatic and asymptomatic cases, with reports following a negative binomial distribution with reporting proportion r and dispersion parameter ϕ). Median estimates are given, with 95% credible intervals in parentheses.

Region	R_0	Reported (%)	Infected (%)
Tahiti	3.7 (2.6-7.8)	11 (5.7-20)	95 (89-99)
Sous-le-vent	3.9 (2.9-7.4)	11 (8-15)	96 (90-100)
Moorea	4.8 (3.1-11)	7.1 (3.9-12)	97 (91-100)
Tuamotu-Gambier	2.9 (2-8.2)	6.9 (3.4-12)	90 (78-97)
Marquises	2.6 (1.8-4)	9.4 (3-21)	87 (73-96)
Australes	3.1 (2-7.5)	17 (8.1-30)	89 (78-97)
