# Supplementary material for: Transmission Dynamics of Zika Virus in Island Populations: A Modelling Analysis of the 2013–14 French Polynesia Outbreak
Source: PLoS Negl Trop Dis. 2016 May 17;10(5):e0004726. doi: 10.1371/journal.pntd.0004726 (PMC4871342; doi:10.1371/journal.pntd.0004726)
Supplement: S3 Table — Estimates for the basic reproduction number, R0; the proportion of infected individuals that were reported as suspected cases at sentinel sites; and the total proportion of the population infected (including both symptomatic and asymptomatic cases, with reports following a negative binomial distribution with reporting proportion r and dispersion parameter ϕ). Median estimates are given, with 95% credible intervals in parentheses. (PDF) [file pntd.0004726.s014.pdf]

Table S3: **Estimated parameters for ZIKV infection when dengue-like prior distributions are used.** Estimates for the basic reproduction number, R_0; the proportion of infected individuals that were reported as suspected cases at sentinel sites; and the total proportion of the population infected (including both symptomatic and asymptomatic cases, with reports following a negative binomial distribution with reporting proportion r and dispersion parameter ϕ). Median estimates are given, with 95% credible intervals in parentheses.

Region	R_0	Reported (%)	Infected (%)
Tahiti	4 (3-6)	11 (5.8-20)	96 (92-98)
Sous-le-vent	4.3 (3.3-5.9)	11 (8.3-14)	97 (92-99)
Moorea	4.8 (3.4-8.3)	7 (3.8-12)	98 (94-99)
Tuamotu-Gambier	3.4 (2.4-6.4)	7 (3.7-12)	91 (84-96)
Marquises	2.8 (2-4.1)	9.5 (3.3-21)	89 (76-95)
Australes	3.4 (2.4-5.3)	17 (8.3-29)	90 (81-95)
