# Supplementary material for: Transmission Dynamics of Zika Virus in Island Populations: A Modelling Analysis of the 2013–14 French Polynesia Outbreak
Source: PLoS Negl Trop Dis. 2016 May 17;10(5):e0004726. doi: 10.1371/journal.pntd.0004726 (PMC4871342; doi:10.1371/journal.pntd.0004726)
Supplement: S4 Table — Estimates for the basic reproduction number, R0; the proportion of infected individuals that were reported as suspected cases at sentinel sites; and the total proportion of the population infected (including both symptomatic and asymptomatic cases, with reports following a negative binomial distribution with reporting proportion r and dispersion parameter ϕ). Median estimates are given, with 95% credible intervals in parentheses. (PDF) [file pntd.0004726.s015.pdf]

Table S4: **Estimated parameters for ZIKV infection when Tahiti serological survey is included in the likelihood.** Estimates for the basic reproduction number, R_0; the proportion of infected individuals that were reported as suspected cases at sentinel sites; and the total proportion of the population infected (including both symptomatic and asymptomatic cases, with reports following a negative binomial distribution with reporting proportion r and dispersion parameter ϕ). Median estimates are given, with 95% credible intervals in parentheses.

Region	R_0	Reported (%)	Infected (%)
Tahiti	1.2 (0.52-1.5)	11 (0.79-46)	67 (63-71)
Sous-le-vent	3.7 (3-5.8)	11 (8.2-14)	96 (92-98)
Moorea	5.6 (3.1-9.9)	7.1 (2.9-13)	97 (92-99)
Tuamotu-Gambier	2.9 (2.2-5)	7 (4-11)	90 (81-95)
Marquises	2.6 (1.9-3.7)	9.4 (2.8-22)	87 (75-95)
Australes	3.1 (2-4.9)	17 (8.7-30)	89 (77-95)
